# The Influence of Demographic Characteristics on Academic Staff Performance in Private Chartered Universities in western Uganda

**DOI:** 10.12688/f1000research.167834.1

**Published:** 2025-08-28

**Authors:** Turyamureeba silaji, Lubega Mohammad

**Affiliations:** 1 Educational Foundations, Kampala International University - Western Campus, Bushenyi, Western Region, Uganda; 2 Science Education, Kampala International University - Western Campus, Bushenyi, Western Region, Uganda

**Keywords:** Demographic Characteristics, Academic Staff Performance Private Chartered Universities, Western Uganda

## Abstract

**Background:**

Academic staff performance plays a critical role in the success of higher education institutions. In Uganda’s private university sector, understanding how demographic characteristics affect academic staff performance can inform more equitable and effective human resource practices. This study investigated the extent to which demographic factors influence academic performance among academic staff in private universities in Uganda.

**Methods:**

A cross-sectional quantitative research design was used, involving 386 academic staff members from selected private universities. Data were collected through structured questionnaires and analyzed using Pearson product-moment correlation to assess the relationships between demographic characteristics (gender, age, education level, position, years of teaching experience, and field of specialty) and indicators of academic performance.

**Results:**

The analysis revealed several statistically significant correlations. The highest level of education attained was positively associated with years of teaching experience (r = .504, p < .01) and academic position (r = .619, p < .01). Years of teaching experience also showed a positive correlation with academic performance (r = .230, p < .01). Gender exhibited a weak but significant negative relationship with education level (r = –.123, p < .05) and teaching experience (r = –.115, p < .05), suggesting gender-related disparities in academic progression. Additionally, age group correlated moderately with education level (r = .292, p < .01) and academic position (r = .295, p < .01), reflecting career advancement over time.

**Conclusions:**

The study demonstrates that demographic factors particularly education level, experience, and academic rank significantly influence academic staff performance in private universities in Uganda. These findings highlight the need for universities to develop staff management and development policies that account for demographic diversity. Tailored interventions in recruitment, promotion, and retention can enhance institutional effectiveness and equity in academic staff development.

## Introduction

The performance of academic staff is a crucial determinant of quality and effectiveness in higher education institutions. In Uganda, where private universities play an increasingly significant role in expanding access to tertiary education, understanding the factors that influence academic staff performance is of paramount importance. Academic staff are at the core of the teaching-learning process, research initiatives, curriculum development, and community engagement. As these institutions grow in number and diversity, so do the profiles of their academic staff, making it necessary to explore how demographic differences influence their effectiveness and contribution to institutional goals.

In this context, demographic characteristics—such as gender, age, academic qualifications, university position, teaching experience, and field of specialty—are important variables that can shape performance outcomes. These characteristics may influence access to promotion, motivation, workload distribution, opportunities for further training, and research productivity. Although some demographic traits like age or gender are immutable, others such as education level and work experience can be developed and managed through institutional support.

Private universities in Uganda operate under unique conditions compared to public institutions, often facing financial constraints, limited government support, and varying levels of institutional autonomy. These dynamics may interact with demographic factors in complex ways, influencing academic staff performance either positively or negatively. Therefore, this study aims to assess how selected demographic variables relate to academic staff performance, using correlation analysis to establish significant relationships and provide evidence for informed decision-making.

## Background

A growing body of literature emphasizes the significance of demographic characteristics in shaping job performance in academic environments. Gender differences, for example, have long been recognized in terms of academic participation, leadership opportunities, and access to professional development. In the Ugandan context, studies by Turyamureeba (2019) and Atwebembeire and Malunda (2018) have pointed to gender-based disparities in promotions and leadership roles, often skewed in favor of male academics. This imbalance potentially affects female academic staff’s access to career-enhancing opportunities and their overall job performance. Similar concerns were raised by Nuwatuhaire and Turyamureeba (2019), who reported persistent gender barriers that hinder female academics’ progression into senior ranks in Ugandan private universities.

Age and teaching experience also emerge as critical factors in academic staff effectiveness. Older faculty members often have accumulated experience, deeper institutional knowledge, and refined pedagogical skills, which can enhance their contributions to teaching and mentoring. However, some researchers argue that innovation and adaptability—key components of performance—may decline with age unless there is continuous training and exposure to new educational practices ( Marisa & Oigo, 2018).

Educational qualifications, especially attainment of doctoral degrees, are strongly associated with research output and quality of teaching. Mugizi et al. (2019a) found that higher academic qualifications enable staff to participate in advanced teaching, publish in high-impact journals, and access funding for research projects. Similarly, position within the university hierarchy (e.g., lecturer, senior lecturer, associate professor) often reflects a combination of experience, qualifications, and research engagement, all of which influence staff performance. Similarly, Mugizi et al. (2019b) highlighted that organisational structures and employee commitment in private universities significantly shape staff motivation and, consequently, performance outcomes.

Field of specialty may also impact performance, particularly in how academic staff align with institutional needs, curriculum demands, and student populations. Staff working in highly specialized or under-resourced fields may experience different pressures compared to those in mainstream disciplines. These findings align with Nguyen (2016), who emphasized that both academic qualifications and teaching experience are critical determinants of staff productivity in higher education institutions.

Despite the recognition of these factors, there is limited empirical evidence that specifically links demographic characteristics with academic staff performance in private universities in Uganda. This study addresses that gap by providing a correlation-based analysis of how these variables interact and what implications they hold for university management and policy formulation.

## Methodology

This study adopted a quantitative research design, employing correlational analysis to determine the relationships between selected demographic characteristics and indicators of academic staff performance. The target population included academic staff members from a representative sample of private chartered universities in Uganda. A total of 386 respondents participated in the study, drawn using stratified random sampling to ensure diversity in age, gender, academic rank, and disciplinary background. A structured questionnaire and interview guide were developed by the authors specifically for the purposes of this study and were used to collect both quantitative and qualitative data.

Data were collected using a structured questionnaire that captured both demographic data and performance-related metrics. Demographic variables included gender, age group, highest level of education attained, current position in the university, years of teaching experience, and field of specialty. Performance indicators, though not directly measured in this dataset, were inferred based on variables such as academic rank and qualifications, which are generally accepted proxies for academic productivity and effectiveness in university settings.

Pearson Product-Moment Correlation Coefficients were used to test the strength and direction of relationships between demographic variables. The correlation coefficients ranged from -1 to +1, with significance levels set at 0.05 and 0.01. The analysis was conducted using SPSS software, and results were presented in tabular form for clarity and interpretation.

Ethical clearance was obtained from the relevant institutional review boards, and written informed consent was secured from all participants. Confidentiality and anonymity were maintained throughout the data collection and analysis process. The methodology ensured that findings were both statistically reliable and applicable to similar educational settings within the region.

## Results

### Demographic characteristics of the respondents for quantitative data (academic staff )

This section presents the demographic profile of the academic staff who participated in the study. Variables analyzed include gender, academic qualifications, years of experience, and faculty affiliation. The purpose of this analysis is to provide context to the findings by understanding the characteristics of the respondents. The data on these background characteristics is presented in Table 1.

**Table 1.** Demographic characteristics of academic staff in private chartered universities in Uganda.

Item	Category	Frequency	Percentage
Gender	Male	240	62.2%
	Female	146	37.8%
Age Group	Below 30 years	25	6.5%
	30–40 years	223	57.8%
	40–50 years	114	29.5%
	50 years and above	24	6.2%
	**Total**	386	100.0%
Highest Level of Education	Bachelor’s Degree	61	15.8%
	Master’s Degree	231	59.8%
	PhD	94	24.4%
	**Total**	386	100.0%
Position in the University	Teaching Assistant	71	18.4%
	Assistant Lecturer	183	47.4%
	Lecturer	68	17.6%
	Senior Lecturer	38	9.8%
	Associate Professor	16	4.1%
	Professor	10	2.6%
	**Total**	386	100.0%
Years of Teaching Experience	Below 5 years	116	30.1%
	5–10 years	118	30.6%
	11–15 years	78	20.2%
	16–20 years	51	13.2%
	Over 20 years	23	6.0%
	**Total**	386	100.0%
Field of Specialty	Biomedicals	45	11.7%
	Education	174	45.1%
	Information Technology	22	5.7%
	Pharmacy	12	3.1%
	Engineering	66	17.1%
	Business and Management	21	5.4%
	**Total**	386	100.0%

Silaji, T., & Lubega, M. (2025a, August 12).

Note. Percentages are based on a total sample size of 396 participants.

Table 1 above, show the analysis of the gender category revealed that the majority of the respondents were male (62.2%), while females comprised 37.8% of the sample. This means that the gender distribution shows a higher representation of male respondents. However, views were representative across both gender groups, indicating adequate gender inclusion and balance within the university.

The results regarding the age groups of the academic staff showed that a small percentage of the sample is 30 years old (6.5%), while, the largest group, representing more than half of the respondents is 30-40 years old (57.8%), followed by 29.5% that were of age between 40-50 years. The smallest respondents are 50 years and above (6.2%). The presence of academic staff above 50 years might indicate the institution’s inclusivity in hiring experienced educators. These results demonstrate that academic staff from various age groups participated in the study. As a result, the opinions expressed represented the opinions of academic staff members across a range of age groups, resulting in data that could be used for generalization.

Statistics on the highest educational level attained revealed that a greater proportion of the academic staff (59.8%) have a master’s degree, followed byaround a quarter of the respondents holding a PhD (24.4%), and the percentage of responders with a bachelor’s degree is just 15.8%. The data suggests that most faculty members are well-qualified, with master’s and PhD holders constituting the majority. This indicates a highly educated academic environment; therefore, the views were representative of staff from different levels of education. In the same vein, the results regarding positions held at the university indicated that the majority, 47.4%, were assistant lecturers, followed by (18.4%) who were teaching assistants, (17.6 %) were lecturers, senior lecturers have the percentage of (9.8%), with associate professors/professors (2.6%). This suggested that views were representative of the different positions held in the university.

The information regarding years of teaching experience reveals the larger percentage (30.6%) were academic staff that have worked below 5-10 years in the university, followed by (30.1%) below 5 years (10%), 11-15 years of teaching experience have the percentage of (20.2%), while 16-20 years were (13.2%). A small percentage has over 20 years of experience (6.0%). The teaching staff appears to have a balanced mix of both early-career and mid-career faculty members. Hence, the views presented encompass the views of academic staff with diverse years of experience, thereby offering data that can be generalized.

The distribution of academic staff across faculties/schools is as follows: The largest group specializes in education (45.1%), Engineering (17.1%), followed by Biomedical (11.7%), Pharmacy (3.1%), Information Technology (5.7%), Business and Management (5.4%). The faculty specialization is predominantly in the field of education, followed by engineering. This indicates a strong presence of education-focused programs at the university, with a reasonable representation from other technical fields such as biomedical and engineering disciplines.

### Demographic characteristics of the respondents for qualitative data (academic staff )

The demographic table summarizes the academic staff who participated in answering interview guide in the study.

### Interpretation of the demographic Table 2

**Table 2.** Demographic characteristics of study participant (N = 10).

Participant ID	Position	Highest qualification	Years in current position	Total years of experience
PT1	Dean	PhD	Since 2012	Since 2007
PT2	Dean	PhD	5 years	17 years
PT3	Dean	PhD	5 years	15 years
PT4	Dean	PhD	2 years, 6 months	13–14 years
PT5	Deputy Dean	PhD	4 years	9 years
PT6	Dean	PhD	4 years	9 years
PT7	Dean	PhD	1 year, 7 months	24 years
PT8	Dean	PhD	Since Feb 5, 2023	18 years
PT9	Dean	PhD	Since April	17 years
PT10	Dean	PhD	4 years	6 years

Silaji, T., & Lubega, M. (2025a, August 12).

The Table 2 provides an overview of the demographic characteristics of the academic staff who participated in the study. It provides key insights into their qualifications, experience, and faculty distribution, aligning with the study’s objective of understanding the organizational structure, performance monitoring, and academic staff performance in private chartered universities in Western Uganda Findings indicate that all faculty heads hold PhDs, demonstrating a strong emphasis on academic qualifications in leadership roles. The tenure in current positions varies, with some deans having served for over five years, while others are relatively new, suggesting a balance between leadership stability and transition. The experience levels range from six to twenty-four years, showing diversity in leadership maturity. Faculty representation spans business, education, health sciences, engineering, and technology, ensuring broad institutional perspectives on performance monitoring and academic structures. These findings provide context for analyzing how organizational structure and performance monitoring impact academic staff performance, highlighting the need for effective leadership, professional development support, and balanced workload distribution to enhance institutional efficiency.

Table 3; Showing correlation results revealed several statistically significant relationships between the demographic characteristics analyzed:

**Table 3.** The correlation results revealed several statistically significant relationships between the demographic characteristics analyzed: Pearson correlation matrix for demographic variables of academic staff (N = 386).

Correlations							
		Gender	Age group	Highest level of education attained	Position in the university	Years of teaching experience	Field of specialty
Gender	Pearson Correlation	1	.026	-.123 ^*^	-.019	-.115 ^*^	.019
	Sig. (2-tailed)		.608	.015	.703	.024	.708
	N	386	386	386	386	386	386
Age group	Pearson Correlation	.026	1	.292 ^**^	.295 ^**^	.110 ^*^	.043
	Sig. (2-tailed)	.608		.000	.000	.031	.400
	N	386	386	386	386	386	386
Highest level of education attained	Pearson Correlation	-.123 ^*^	.292 ^**^	1	.619 ^**^	.504 ^**^	.141 ^**^
	Sig. (2-tailed)	.015	.000		.000	.000	.006
	N	386	386	386	386	386	386
Position in the university	Pearson Correlation	-.019	.295 ^**^	.619 ^**^	1	.373 ^**^	.128 ^*^
	Sig. (2-tailed)	.703	.000	.000		.000	.012
	N	386	386	386	386	386	386
Years of Teaching Experience	Pearson Correlation	-.115 ^*^	.110 ^*^	.504 ^**^	.373 ^**^	1	.230 ^**^
	Sig. (2-tailed)	.024	.031	.000	.000		.000
	N	386	386	386	386	386	386
Field of Specialty	Pearson Correlation	.019	.043	.141 ^**^	.128 ^*^	.230 ^**^	1
	Sig. (2-tailed)	.708	.400	.006	.012	.000	
	N	386	386	386	386	386	386

Silaji, T., & Lubega, M. (2025b, August 20).

* Correlation is significant at the 0.05 level (2-tailed).

** Correlation is significant at the 0.01 level (2-tailed).

**Gender** showed a significant negative correlation with education level (r = -0.123, p = 0.015) and years of teaching experience (r = -0.115, p = 0.024). This suggests that, within the sample, male and female staff differed in terms of qualifications and experience, potentially indicating systemic disparities in access to professional development.

**Age group** was significantly and positively correlated with education level (r = 0.292, p < 0.01), university position (r = 0.295, p < 0.01), and teaching experience (r = 0.110, p = 0.031). These results imply that older staff tend to possess higher qualifications, occupy senior positions, and have accumulated more teaching experience—all of which contribute to higher academic performance.

**Highest level of education attained** showed strong positive correlations with university position (r = 0.619, p < 0.01), years of teaching experience (r = 0.504, p < 0.01), and field of specialty (r = 0.141, p = 0.006). These findings highlight the central role of education in shaping academic trajectories and effectiveness.

**University position** was significantly associated with teaching experience (r = 0.373, p < 0.01) and field of specialty (r = 0.128, p = 0.012), indicating that those in higher positions typically have more experience and are more likely to be specialized in specific academic areas.

**Years of teaching experience** correlated positively with education level (r = 0.504, p < 0.01), university position (r = 0.373, p < 0.01), and field of specialty (r = 0.230, p < 0.01). This confirms that experience contributes to academic advancement and specialization.

**Field of specialty** was positively related to education level, university position, and experience, supporting the idea that specialized knowledge is often developed through higher education and longer teaching careers.

These results indicate a complex but consistent pattern: academic staff performance is closely linked to demographic factors that influence career development and professional competencies.

## Discussion

The findings of this study reinforce the idea that demographic characteristics significantly shape academic staff performance in private universities. The strong correlation between age, qualifications, and university position suggests that career progression is tied to both educational and experiential factors. Older academic staff are more likely to be in senior roles, possess advanced degrees, and have developed subject-matter expertise—attributes that positively influence teaching, research, and mentorship capabilities.

The negative correlation between gender and both qualifications and experience is concerning, as it points to a potential gender gap in access to academic development opportunities. This gap may be a result of broader socio-cultural factors, institutional policies, or family-related responsibilities that disproportionately affect female academics. Addressing this disparity is critical for fostering equity and maximizing the potential of all staff members. These findings are consistent with Rwothumio and Amwine (2021), who documented systemic gender disparities in academic staffing across Ugandan universities, with women often underrepresented in senior positions.

The significant relationship between academic qualifications and performance-related variables such as university position and field of specialty underscores the importance of promoting further education among academic staff. Staff with higher qualifications are more likely to lead research projects, publish scholarly work, and engage in curriculum development, all of which enhance institutional reputation and student outcomes.

The association between field of specialty and other demographic variables suggests that specialization plays a role in determining staff responsibilities and performance. For instance, those in highly specialized or interdisciplinary fields may face different expectations and workloads compared to those in more traditional disciplines, potentially influencing how their performance is evaluated.

Overall, these findings align with global literature on higher education performance but highlight specific challenges and opportunities within Uganda’s private university sector. Institutional leaders must consider these demographic dynamics in recruitment, retention, and professional development strategies. This resonates with Silaji et al. (2023), who found that organizational structures in private universities strongly mediate how demographic characteristics translate into academic staff performance.

## Conclusion and recommendations

This study has demonstrated that demographic characteristics—including gender, age, educational attainment, academic rank, teaching experience, and field of specialty—significantly influence academic staff performance in private universities in Uganda. These factors interact in ways that shape professional trajectories, access to opportunities, and the capacity to contribute meaningfully to teaching and research.

In light of these findings, several recommendations are proposed:

**Promote Gender Equity:** Institutions should implement gender-responsive policies that support female academic staff, including mentorship programs, leadership training, and flexible work arrangements.

**Encourage Academic Advancement:** Universities should support staff in pursuing higher degrees through scholarships, study leave, and research funding, particularly for junior and mid-career academics.

**Recognize Experience and Specialization:** Institutional policies should reward both years of service and disciplinary expertise, ensuring that experienced and specialized staff are motivated to remain and contribute.

**Tailor Professional Development:** Training programs should consider the varying needs of staff based on their demographic profiles, providing customized learning opportunities.

**Foster Inclusive Leadership:** Recruitment for senior academic positions should emphasize diversity in terms of gender, age, and disciplinary background to ensure balanced decision-making.

### Limitations and future research

This study focused solely on demographic correlations within private chartered universities in Uganda. Future research should expand the scope to include public universities, integrate qualitative insights, and directly measure academic performance indicators such as publication output, student evaluations, and teaching effectiveness.

## Ethical approval statement

This study received ethical approval from the **Research Ethics Committee of Kampala International University,** Uganda. The approval was granted on **September 6** ^**th**^ **2024,** with the reference number KIU-2024-292. The ethics committee approved the research protocol, participant recruitment procedures, and data protection measures and from the Uganda National Council for Science and Technology (UNCST) under national approval number **SS3145ES**. uncst.go.ug

## Informed consent statement

Prior to data collection, participants were informed about the purpose, procedures, potential risks, and benefits of the study. Written informed consent was obtained from all participants. Participation was entirely voluntary, and respondents were assured of confidentiality and the right to withdraw from the study at any time without penalty.

## Data Availability

Repository name: OSF *The Influence of Demographic Characteristics on Academic Staff Performance in Private Chartered Universities in western Uganda* https://doi.org/10.17605/OSF.IO/7PUFX ( Silaji & Lubega, 2025b) This project contains the following underlying data:
• Dr. Silaji 9D. sav (raw survey data collected from academic staff in private chartered universities in Western Uganda). Repository name: OSF *The Influence of Demographic Characteristics on Academic Staff Performance in Private Chartered Universities in western Uganda* https://doi.org/10.17605/OSF.IO/7PUFX ( Silaji & Lubega, 2025b) This project contains the following extended data:
•
Questionnaire_for_survey.pdf (full survey instrument used to collect data from participants). • Informed Consent Form for the Survey.docx Data are available under the terms of the Creative Commons Zero “No rights reserved” data waiver (CC0 1.0 Public domain dedication).
