# Supplementary material for: The Influence of Demographic Characteristics on Academic Staff Performance in Private Chartered Universities in western Uganda
Source: F1000Res. 2026 Mar 25;14:833. Originally published 2025 Aug 28. [Version 4] doi: 10.12688/f1000research.167834.4 (PMC12946698; doi:10.12688/f1000research.167834.4)
Supplement: Peer review history

# Reviewer report: Reviewer response for version 4

**DOI:** 10.5256/f1000research.197206.r470527

**Published:** 2026-04-07

**Authors:** Regis Misheal Muchowe

**Affiliations:** 1 Zimbabwe Open University, Harare, Zimbabwe

Good day,

 Thank you for considering my previous comments. However, on the issue of statistical analysis, I feel there is need for further revisions. My previous comment was that you cannot use Pearson regression for all the variables. Some are continuous, some are numerical, some are categorical and some are ordinal. I recommended some tests that can be used for different variables. I did not see a response or justification on why this section has not been improved according to appropriate tests. I believe if this section is improved your paper will be of impact.

Is the work clearly and accurately presented and does it cite the current literature?

Yes

If applicable, is the statistical analysis and its interpretation appropriate?

Partly

Are all the source data underlying the results available to ensure full reproducibility?

Yes

Is the study design appropriate and is the work technically sound?

Yes

Are the conclusions drawn adequately supported by the results?

Yes

Are sufficient details of methods and analysis provided to allow replication by others?

Yes

Reviewer Expertise:

Human resources management, employee performance, employee motivation, HR analytics, AI and HRM.

I confirm that I have read this submission and believe that I have an appropriate level of expertise to confirm that it is of an acceptable scientific standard, however I have significant reservations, as outlined above.

# Reviewer report: Reviewer response for version 3

**DOI:** 10.5256/f1000research.189729.r460285

**Published:** 2026-02-26

**Authors:** Regis Misheal Muchowe

**Affiliations:** 1 Zimbabwe Open University, Harare, Zimbabwe

This is a timely research. However, I feel the following corrections can strengthen your research output and impact:

 1. On the abstract. The first seentence needs to alert the reader of the problem that stimulated your research.

 2. On the results (abstract): Abstract should be brief, I recommend you to only summarise results of demographic factors and the dependent variable, and reserve other results such as demographic data v. demographic data, for the other sections in the paper.

 3. Add theoretical framework on your literature review.

 4. Your introduction section is not supported by references. You need to substantiate your claims with references.

 5.Which SPSS software version was used.

 6. Demographic factors are different not all require Pearson test. Pearson test is relevant where the two variables tested are continuous eg Age and performance. However, for gender you can use Independent T-test, Pearson for Age, Years-Pearson, Field and Education use Anova.

 7. Discussions should also be based on theories that you would have discussed in the literature so that readers are aware of how theory is expanded or substantiated by this research.

 8. On limitations you did not elucidate the fact that the data was self-reported.

 9. Finally, was the questionnaire adopted or adapted. If adopted from who?

Is the work clearly and accurately presented and does it cite the current literature?

Yes

If applicable, is the statistical analysis and its interpretation appropriate?

Partly

Are all the source data underlying the results available to ensure full reproducibility?

Yes

Is the study design appropriate and is the work technically sound?

Yes

Are the conclusions drawn adequately supported by the results?

Yes

Are sufficient details of methods and analysis provided to allow replication by others?

Yes

Reviewer Expertise:

Human resources management, employee performance, employee motivation, HR analytics, AI and HRM.

I confirm that I have read this submission and believe that I have an appropriate level of expertise to confirm that it is of an acceptable scientific standard, however I have significant reservations, as outlined above.

# Response

**Published:** 2026-02-27

**Authors:** Turyamureeba silaji

**Affiliations:** Foundations, Kampala International University - Western Campus, Bushenyi, Western Region, Uganda

We sincerely thank the reviewer for the thorough, constructive, and insightful feedback provided on our manuscript. Your comments have significantly strengthened the clarity, methodological rigor, and theoretical grounding of this study. In particular, your guidance on refining the abstract, strengthening the statistical justification, incorporating a clearer theoretical framework, and improving methodological transparency has greatly enhanced the overall quality and scholarly contribution of the paper.

 We deeply appreciate the time, expertise, and thoughtful engagement you invested in reviewing our work. Your recommendations have helped us improve both the scientific robustness and practical relevance of the manuscript.

# Reviewer report: Reviewer response for version 3

**DOI:** 10.5256/f1000research.189729.r425048

**Published:** 2025-10-30

**Authors:** Denok Sunarsi

**Affiliations:** 1 Universitas Pamulang, Tangerang, Banten, Indonesia

I would like to commend the authors for their thoughtful and thorough response to my feedback. The revisions made, particularly the inclusion of interpretive analysis on how demographic characteristics such as gender, age, education, academic position, teaching experience, and field of expertise influence academic staff performance, have significantly strengthened the manuscript. This enhancement ensures that the study aligns more closely with its title and objectives, enriching its contribution to the literature, especially in the context of private universities in Uganda.

 The added references, such as Nuwatuhaire & Turyamureeba (2019), Rwothumio & Amwine (2021), and Mugizi et al. (2019a), further support the authors' arguments, and the connections drawn between demographic factors and staff performance are well articulated. Overall, the revisions have addressed my concerns and improved the manuscript

Is the work clearly and accurately presented and does it cite the current literature?

Yes

If applicable, is the statistical analysis and its interpretation appropriate?

Partly

Are all the source data underlying the results available to ensure full reproducibility?

Partly

Is the study design appropriate and is the work technically sound?

Partly

Are the conclusions drawn adequately supported by the results?

Partly

Are sufficient details of methods and analysis provided to allow replication by others?

Partly

Reviewer Expertise:

My area of expertise includes higher education management, academic staff development, and quantitative research methods, particularly in the context of demographic influences on professional performance

I confirm that I have read this submission and believe that I have an appropriate level of expertise to confirm that it is of an acceptable scientific standard.

# Response

**Published:** 2025-10-30

**Authors:** Turyamureeba silaji

**Affiliations:** Foundations, Kampala International University - Western Campus, Bushenyi, Western Region, Uganda

I would also like to sincerely thank the reviewer for their insightful and constructive feedback, which greatly enhanced the quality and clarity of my manuscript. Your detailed observations guided meaningful revisions, particularly in expanding the interpretive analysis of how demographic characteristics—such as gender, age, education, academic position, teaching experience, and field of expertise—influence academic staff performance. Your engagement and scholarly perspective helped ensure that the study more effectively aligns with its title, objectives, and overall contribution to understanding academic staff dynamics in private universities in Uganda.

 I am especially grateful for your recognition of the revisions made and for acknowledging the integration of additional references such as Nuwatuhaire & Turyamureeba (2019), Rwothumio & Amwine (2021), and Mugizi et al. (2019a), which strengthened the theoretical and empirical foundation of the paper. Your professional feedback not only improved this particular study but also contributed to my ongoing academic growth. Thank you once again for your valuable time, expertise, and encouragement.

# Reviewer report: Reviewer response for version 2

**DOI:** 10.5256/f1000research.188536.r423489

**Published:** 2025-10-14

**Authors:** Denok Sunarsi, Rofiq Noorman Haryadi

**Affiliations:** 1 Manajement, STEBIS Bina Mandiri, Bogor, West Java, Indonesia; 2 Universitas Pamulang, Tangerang, Banten, Indonesia

The article examines the influence of demographic characteristics on academic staff performance in private universities in Uganda, using a quantitative cross-sectional study design. The study found significant correlations between factors such as age, gender, education level, and teaching experience, and how these influence academic staff performance. The findings emphasize the need for universities to consider demographic diversity in their policies.

 However, there are areas that need improvement. First, while the correlation analysis is robust, the authors should expand their interpretation to clearly show how these demographic factors actively shape academic performance, rather than just identifying statistical associations. In addition, the methodology could benefit from considering causal analysis to provide a more comprehensive understanding. The discussion around gender disparities in qualifications and experience should be further explored, including underlying institutional or cultural factors. Finally, the recommendations for policy changes could be more specific, offering concrete examples of how private universities can address gender inequality and promote academic advancement for all staff. Addressing these points would make the article scientifically sound and more actionable for educational institutions

Is the work clearly and accurately presented and does it cite the current literature?

Yes

If applicable, is the statistical analysis and its interpretation appropriate?

Partly

Are all the source data underlying the results available to ensure full reproducibility?

Partly

Is the study design appropriate and is the work technically sound?

Partly

Are the conclusions drawn adequately supported by the results?

Partly

Are sufficient details of methods and analysis provided to allow replication by others?

Partly

Reviewer Expertise:

My area of expertise includes higher education management, academic staff development, and quantitative research methods, particularly in the context of demographic influences on professional performance

We confirm that we have read this submission and believe that we have an appropriate level of expertise to confirm that it is of an acceptable scientific standard, however we have significant reservations, as outlined above.

# Response

**Published:** 2025-10-14

**Authors:** Turyamureeba silaji

**Affiliations:** Foundations, Kampala International University - Western Campus, Bushenyi, Western Region, Uganda

**Author Response Letter to Reviewer**

**Manuscript Title:** *The Influence of Demographic Characteristics on Academic Staff Performance in Private Chartered Universities in Western Uganda*

**Authors:** Turyamureeba Silaji & Lubega Mohammad

**Journal:** *F1000Research*

**Date:** 14/10/2025

**Dear Reviewer,**

 We would like to sincerely thank you for your thorough and insightful review of our manuscript. Your constructive comments have been extremely valuable in improving the clarity, depth, and practical relevance of our work. We carefully considered each point and made comprehensive revisions accordingly. Below, we outline how your feedback was addressed in the revised version.

**1. Interpretation Beyond Statistical Associations**

**Reviewer Comment:**

*While the correlation analysis is robust, the authors should expand their interpretation to clearly show how these demographic factors actively shape academic performance, rather than just identifying statistical associations.*

**Response:**

 We fully agree with this observation. The **Results** and **Discussion** sections have been expanded to explain *how* demographic factors influence academic staff performance rather than merely reporting correlations. For example, we now elaborate on how higher qualifications enhance research productivity and leadership readiness, how age and experience improve teaching and mentoring capacity, and how gender disparities affect access to professional development opportunities. These interpretations are supported with current literature, including Marisa & Oigo (2018), Mugizi et al. (2019a, b), Rwothumio & Amwine (2021), and Silaji et al. (2023). This revision ensures that the findings reflect both statistical and practical significance.

**2. Methodological Consideration of Causality**

**Reviewer Comment:**

*The methodology could benefit from considering causal analysis to provide a more comprehensive understanding.*

**Response:**

 We acknowledge this valuable point. While the current study employed a cross-sectional correlational design, we have added a statement under the **Limitations and Future Research** section acknowledging this limitation. We also recommend that future research adopt **longitudinal or causal modeling approaches**, such as structural equation modeling (SEM), to establish cause–effect relationships between demographic factors and academic performance. This addition clarifies the scope of our design and enhances methodological transparency.

**3. Expanded Discussion on Gender Disparities**

**Reviewer Comment:**

*The discussion around gender disparities in qualifications and experience should be further explored, including underlying institutional or cultural factors.*

**Response:**

 We appreciate this important suggestion. The **Discussion** section has been revised to include a deeper analysis of the institutional and socio-cultural factors contributing to gender disparities in academic qualifications and experience. We now discuss how family-care responsibilities, institutional bias in promotion systems, and limited mentorship opportunities constrain female academics’ career progression. This discussion aligns with evidence from Nuwatuhaire & Turyamureeba (2019) and Rwothumio & Amwine (2021), providing a more contextually grounded interpretation of gender inequality in Ugandan private universities.

**4. Strengthening of Policy Recommendations**

**Reviewer Comment:**

*The recommendations for policy changes could be more specific, offering concrete examples of how private universities can address gender inequality and promote academic advancement for all staff.*

**Response:**

 We fully agree. The **Recommendations** section has been rewritten to include actionable and context-specific policy strategies. These include:

- Establishing **gender-responsive mentorship and leadership programs** for female academics.
- Providing **study-leave and research grants** to support postgraduate training and professional growth.
- Implementing **transparent, merit-based promotion criteria** to ensure equity.
- Developing **inclusive leadership pathways** that value diversity across age, gender, and disciplines.
  These refinements make the recommendations more practical and directly relevant to institutional policy and management.

**5. Overall Clarity and Alignment**

**Reviewer Comment:**

*Addressing these points would make the article scientifically sound and more actionable for educational institutions.*

**Response:**

 We have reviewed and refined the **Abstract**, **Discussion**, and **Conclusion** sections to ensure consistency with the expanded analyses and recommendations. The revised conclusion now synthesizes the findings, highlights the practical implications for university policy, and outlines directions for future research. Language and structure were improved throughout to enhance readability and coherence.

**Summary of Key Revisions**

- Expanded interpretation linking demographic factors directly to academic performance.
- Acknowledged methodological limitations and suggested future causal research designs.
- Broadened gender analysis with institutional and cultural context.
- Strengthened recommendations with concrete, actionable policy strategies.
- Refined abstract, discussion, and conclusion for clarity and alignment.

**Closing Statement**

 We are deeply grateful for your thoughtful and constructive review. Your comments have guided substantial improvements that enhanced both the scientific and practical quality of this paper. We believe the revised version now presents a more comprehensive, methodologically sound, and policy-relevant contribution to the literature on academic staff performance and demographic diversity in higher education.

 With sincere appreciation,

**Turyamureeba Silaji**

 Corresponding Author:

*silaji.turyamureeba@studwc.kiu.ac.ug*

Kampala International University

# Reviewer report: Reviewer response for version 1

**DOI:** 10.5256/f1000research.184977.r410120

**Published:** 2025-09-18

**Authors:** Abd Al-Aziz Al-refaei

**Affiliations:** 1 Lincoln University College, Petaling Jaya, Selangor, Malaysia

The paper is valuable in providing insights into The Influence of Demographic Characteristics on Academic Staff Performance in Private Chartered Universities in western Uganda. A few improvements would be helpful to enhance this work

 Title: the title highlighted the influence of Demographic Characteristics on Academic Staff Performance in Private Chartered Universities in western Uganda, but unfortunately, the study confined on examine correlations between the demographic characteristics, and has not examine how these demographic characteristics influence the academic staff performance?

 I recommend the authors to expand their analysis to include how these demographic characteristics influence the academic staff performance? (these has been presented in the literature review but has not been examined the analysis section).

 In the background what has been done is valuable. In addition, it is recommended to present what distinguishes your study from another previous study.

Is the work clearly and accurately presented and does it cite the current literature?

Yes

If applicable, is the statistical analysis and its interpretation appropriate?

Partly

Are all the source data underlying the results available to ensure full reproducibility?

Partly

Is the study design appropriate and is the work technically sound?

Yes

Are the conclusions drawn adequately supported by the results?

Partly

Are sufficient details of methods and analysis provided to allow replication by others?

Partly

Reviewer Expertise:

HRM, HRD, Higher education service quality, leadership, data analysis (SEM).

I confirm that I have read this submission and believe that I have an appropriate level of expertise to confirm that it is of an acceptable scientific standard, however I have significant reservations, as outlined above.

# Response

**Published:** 2025-09-20

**Authors:** Turyamureeba silaji

**Affiliations:** Foundations, Kampala International University - Western Campus, Bushenyi, Western Region, Uganda

**Response to Reviewer**

 Dear Reviewer,

 We sincerely thank you for the time and effort you dedicated to reviewing our manuscript titled:

**“The Influence of Demographic Characteristics on Academic Staff Performance in Private Chartered Universities in Western Uganda.”**

 Your insightful feedback has greatly improved the quality of our work. Below, we provide our response to your key comment:

**Reviewer Comment:**

*The title highlighted the influence of Demographic Characteristics on Academic Staff Performance in Private Chartered Universities in western Uganda, but unfortunately, the study confined on examining correlations between the demographic characteristics, and has not examined how these demographic characteristics influence the academic staff performance. I recommend the authors to expand their analysis to include how these demographic characteristics influence the academic staff performance. (These have been presented in the literature review but have not been examined in the analysis section).*

**Author Response:**

 We agree with your observation that the initial manuscript emphasized correlations without sufficiently interpreting how demographic characteristics influence academic staff performance. We have therefore revised the manuscript to address this concern.

- **Results section (pp. 6–7, after Table 3):** We added interpretive text that explains how gender, age, educational qualifications, academic position, teaching experience, and field of specialty actively shape academic staff performance. For example, we now show that gender disparities influence access to qualifications and leadership opportunities (Nuwatuhaire & Turyamureeba, 2019; Rwothumio & Amwine, 2021), and that doctoral qualifications enhance research and curriculum contributions (Mugizi et al., 2019a).
- **Discussion section (pp. 7–8):** We expanded the discussion by inserting two new passages. These explain how demographic factors act as determinants of performance rather than merely background characteristics, linking the results to the literature cited in the study (e.g., Marisa & Oigo, 2018; Turyamureeba, 2019; Silaji et al., 2023).

These revisions ensure that the analysis now matches the title and objectives of the study, and we believe they strengthen the manuscript’s contribution to the literature on academic staff performance in Uganda’s private universities.

 We are grateful for your constructive feedback, which helped us significantly improve our paper.

 With kind regards,

 Turyamureeba Silaji & Lubega Mohammad
